# Efficacy, safety, and feasibility of volumetric modulated arc therapy for synchronous bilateral breast cancer management

**DOI:** 10.3389/fonc.2022.967479

**Published:** 2022-08-18

**Authors:** Stanislas Quesada, Pascal Fenoglietto, Sophie Gourgou, Claire Lemanski, Roxana Draghici, Norbert Ailleres, Jessica Prunaretty, David Azria, Céline Bourgier

**Affiliations:** ^1^ Faculty of Medicine, University of Montpellier, Montpellier, France; ^2^ Department of Radiation Oncology, Montpellier Cancer Institute (ICM), Montpellier, France; ^3^ Institute of Cancer Research of Montpellier (IRCM), Montpellier, France

**Keywords:** bilateral breast cancer, radiotherapy, VMAT, treatment planning, cancer care, dosimetric analysis, treatment outcome

## Abstract

**Purpose:**

Volumetric Modulated Arc Therapy (VMAT) exhibits potent advantages regarding target volume coverage and protection of organs at risk, notably in the context of anatomical constraints. Nevertheless, reports concerning VMAT for the treatment of synchronous bilateral breast cancers (SBBC) have been scarce to date. As such, we conducted this observational study to assess efficacy, safety and feasibility of VMAT in SBBC.

**Materials and Methods:**

From August 2011 to December 2017, 54 consecutive patients with SBBC with or without axillary nodes involvement underwent a treatment protocol containing radiotherapy using VMAT. A total dose (TD) of 52.2Gy in 29 fractions was delivered to breast and internal mammary chain (IMC) nodes Planning Target Volume (PTV) plus, if applicable, a TD of 49.3Gy in 29 fractions to the supra- and infra-clavicular nodes PTV and a TD of 63.22Gy in 29 fractions to tumor boost PTV. Lungs, heart, esophagus, trachea, liver, thyroid and spinal cord were considered as organs at risk. VMAT feasibility and organ at risk sparing were evaluated by treatments planning of the 20 first enrolled patients. Tolerance and patients’ outcome were prospectively monitored by acute/late toxicities records and by the analysis of overall survival (OS), locoregional recurrence-free survival (LRFS) and recurrence-free survival (RFS).

**Results:**

Breast, supraclavicular nodes and boost PTV coverage was adequate with at least 98% of PTV encompassed by more than 95% of the prescribed dose. Less than 90% of IMC PTV was encompassed by 95% of the prescribed dose. Mean lung dose was 12.3Gy (range: 7.7 – 18.7); mean heart dose was 10.7Gy (range: 6.2 – 22.3). Concerning acute toxicities, only 2 patients experienced grade 3 skin toxicity (3.7%) and only 1 patient developed grade 1 pneumonitis. After a median follow-up of 5.3 years, grade 2 fibrosis and/or shrinking was observed in 5 patients (10%), and grade 3 fibrosis in 1 patients (2%). The 5-year LRFS-rate, RFS-rate and OS were 98% [95% CI= 86.12-99.70%], 96% [95% CI= 84.63-98.96%] and 100%, respectively.

## Introduction

Breast cancer (BC) is the most frequently diagnosed cancer worldwide (more than 2 million cases diagnosed in women worldwide in 2018), and also the most frequent cause of cancer-related death in women (1). Synchronous bilateral breast cancer (SBBC), defined as the presence of at least two malignant lesions occurring simultaneously in both breasts, accounts for ≈2% of all BC. Although SBBC represents only a small percentage of all BC, due to the high BC incidence, every year approximately 40,000 new cases of SBBC are detected (2). BC multimodal management includes surgery, systemic therapies, and radiation therapy (RT). RT is mandatory after breast-conserving surgery, and indicated after mastectomy in patients with locally advanced BC (3–5). However, currently, there is no specific recommendation for SBBC that is managed by following the guidelines for unilateral BC. In SBBC, Intensity Modulated Radiation Therapy (IMRT), either through fixed-field or preferably with volumetric modulated arc therapy (VMAT) represents a relevant alternative to 3D-conformal RT (6, 7). Indeed, VMAT has been shown to provide adequate target volume coverage while sparing the organs at risk (OAR) (8, 9). Some reports described the use of VMAT for SBBC and other complex situations (10–12). Nevertheless, only a few small retrospective cohorts (*i.e.* less than 25 patients) reported their experience on VMAT use in patients with SBBC (13–15). At our center, VMAT is used for the management of complex BC and for SBBC. The aim of this monocentric observational study was to assess the dosimetric feasibility, efficacy, safety and long-term outcome of VMAT for SBBC management.

## Materials and methods

### Patients and treatment protocol

From August 2011 to December 2017, all consecutive patients which received VMAT (Varian Medical systems, Palo Alto, USA) for SBBC at the Institut Regional du Cancer de (Montpellier, France) were included. During this period, VMAT and 3D RT were used in our center. VMAT was indicated over 3D because of node irradiation necessity and/or anatomical constraints (*e.g.*, *pectus excavatum*). This retrospective observational study was approved by the local Ethics Committee. The inclusion criteria were as follows: older than 18 years of age, Eastern Cooperative Oncology Group (ECOG) score ≤2, and histologically confirmed SBBC. Breast surgery (either mastectomy or breast conserving surgery), neoadjuvant or adjuvant chemotherapy, targeted therapies and endocrine therapies were delivered according to the clinical practice guidelines.

### Radiation therapy process

Patient positioning/immobilization for VMAT, anatomy data acquisition, target volume definition, organs at risk evaluation, dose prescription, treatment planning and dosimetric analysis are reported in **Supplementary Data**. Treatment plans of the first 20 patients were used for dosimetric analysis (*i.e.*, dose volume distribution for breasts, nodes and organs at risk) as an evaluation of feasibility of VMAT for SBBC.

### Radiation-related toxicities

Acute radiation-related toxicities were recorded every week during VMAT and late toxicities were recorded every 6 months after RT completion. Between end of VMAT and medical consultation at 6-month, supplementary medical evaluation was performed upon patient’s request. Both acute and late toxicities were clinically assessed and graded according to the Common Terminology Criteria for Adverse Events (CTCAE) v4.0; respiratory, cardiac, esophageal and cutaneous toxicities were systematically evaluated. For each patient, the highest toxicity score reached was retained. Cardiac toxicity was defined as any cardiac event such as ischemia, arrhythmia or heart failure. Late cutaneous toxicities were subdivided according to the following subscales: fibrosis, hyperpigmentation, shrinking, telangiectasia and breast edema. Skin toxicities were evaluated both at patient and breast scales, as two distinct protocols (*i.e.*, surgery and radiation therapy) were possible for a given patient, with possible impacts on sequelae. Data on the patients’ outcome and late toxicities were collected up to December 2020.

### Statistical analysis

The median follow-up was estimated using the inverse Kaplan-Meier method. The Kaplan-Meier method was used for estimating recurrence-free survival (RFS: calculated from the beginning of treatment until recurrence or death), locoregional-free survival (LRFS: calculated from the beginning of treatment until locoregional recurrence or death), and overall survival (OS: calculated from the beginning of treatment until the date of death). For patients with metastatic SBBC at diagnosis, RFS corresponded to the progression-free survival (PFS). Data were analyzed both at the patient (n=54) and at the tumor (n=108) levels.

To identify predictive factors of skin toxicities (acute or late), patient and tumor characteristics [age, tumor stage (according to the TNM classification), Scaff-Bloom-Richardson (SBR) grade, hormonal receptor positivity, HER2 amplification, histology] and treatment characteristics [surgery (lumpectomy or mastectomy), systemic therapies, total irradiated volume, PTV, boost volume/PTV ratio] were analyzed. The Chi-square test was used to determine the correlation between categorical variables (or the Fisher’s exact test if the expected frequencies were lower than 5). The Spearman’s correlation test was used to assess the correlation between ordinal variables. The Wilcoxon test was used to study the correlation between nominal qualitative variables and continuous quantitative variables. Logistic regression was performed to identify factors related to skin toxicity; the p-values ​​of the logistic regression were computed with the likelihood ratio test. Each Odd Ratio (OR) is presented with its 95% confidence interval (CI). For multivariate analysis, variables were selected using the backward method: variables with the largest p-value were removed one by one until only significant variables (at the 5% level) remained. All statistical tests were two-sided and the significance level was set at 5% (p <0.05).

## Results

### Patients and treatment characteristics

From August 2011 to December 2017, 54 consecutive patients were prospectively enrolled and followed (median follow-up = 5.3 years [min-max=0.46-8.74]). Characteristics of patients/tumors and treatments provided are listed in **Tables 1**, **2**, respectively. Briefly, 39 patients (72.1%) had early bilateral BC (EBC), 1 (1.8%) locally advanced bilateral BC (LABC), and 10 (18.5%) both LABC and EBC. Four patients (7.4%) had at least one metastasis at diagnosis. More than half of patients received neo-adjuvant or adjuvant chemotherapy.

**Table 1:** Characteristics of patients (n=54) and breast cancer lesions (n=108).

**Age** (years), median (range)	63 (35-82)
**Histological type**, breast cancer lesions’ number (%)	
Invasive ductal carcinoma	79 (73.1%)
Invasive lobular carcinoma	11 (10.2%)
Other types	6 (5.6%)
*In situ* carcinoma	12 (11.1%)
**Scarff-Bloom-Richardson grade**, breast cancer lesions’ number (%)	
I	27 (27.8%)
II	53 (54.6%)
III	17 (17.6%)
Missing	11
**Hormone receptor expression**, breast cancer lesions’ number (%)	
Negative	7 (7.2%)
Positive	90 (92.8%)
Missing	11
**HER2 amplification** (breast cancer lesions’ number, %)	
Negative	87 (89.7%)
Positive	10 (10.3%)
Missing	11
**Pathological T stage** (breast cancer lesions’ number, %)	
pT0/ypT0	14 (13.2%)
pT1/ypT1	66 (62.3%)
pT2/ypT2	25 (18.9%)
pT3/ypT3	5 (4.7%)
pT4/ypT4	1 (0.9%)
Unknown	2
**Pathological nodes status,** breast cancer lesions’ number (%)	
Negative (pN0/ypTN0)	75 (79.8%)
Positive (pN+/ypTN+)	19 (20.2%)
Unknown	14
**Metastases at diagnosis**, patients’ number (%)	4 (7.4%)

**Table 2:** Treatment characteristics of patients (n=54) and breast cancer lesions (n=108).

**Primary surgery**, breast cancer lesions’ number (%)	
Breast conserving surgery	92 (85.2%)
Mastectomy	14 (13%)
No surgery	2 (1.8%)
**Axillary lymph node staging**	
Sentinel node biopsy	66 (61.1%)
Axillary node dissection	30 (27.8%)
None	12 (11.1%)
**Radiotherapy radiation fields**, breast number (%)	
Exclusive mammary gland	15 (13.9%)
Mammary gland and simultaneous integrated tumor boost (SIB)	83 (76.8%)
Regional lymph nodes	32 (29.6%)
Chest wall	10 (9.3%)
**Irradiated volumes** (cm³), mean value (range)	
Planning Target Volume (PTV)	945 (262-2421)
SIB volume	113 (11-288)
SIB/PTV ratio in %	12 (4-32)
Total irradiated volume per patient (cm³), mean value (range)	1880 (544-4811)
**Neoadjuvant chemotherapy,** patients’ number (%)	
Yes	10 (18.5%)
No	44 (81.5%)
**Adjuvant chemotherapy**, patients’ number (%)	
Yes	18 (33.4%)
No	36 (66.6%)
**Post-radiotherapy systemic therapy** patients’ number (%)	
Endocrine therapy (tamoxifen and/or aromatase inhibitor)	51 (94.4%)
Trastuzumab	6 (11.2%)

Bilateral and unilateral breast conserving surgeries were performed in 43 and in 6 patients, respectively (n=92 breasts). Regarding treatment planning, 47 patients (n=83 breasts) received mammary gland irradiation with simultaneous integrated boost (SIB) in at least one breast, and 22 patients (n=32 breasts) received also regional node irradiation in at least one breast. The mean PTV was 945 cm^3^ (range, 262 – 2421); when SIB was performed, the mean SIB/PTV_SIB ratio was 12% (range, 4 – 32).

The analysis of the first 20 treatment plans (**Supplementary Data, Table 1**) showed adequate PTV coverage: V95%=98.9% for both breast sides, and >98% for regional nodes (**Supplementary Data, Figure 1**). The mean lung doses were 12.0 Gy (left lung) and 12.7 Gy (right lung). The mean heart dose was 10.7 Gy for the entire cohort: 8.8Gy with only breast irradiation and 12.5Gy with associated IMC node irradiation (**Supplementary Data, Figure 2**).

### Acute and late toxicities

Acute toxicities: 35 (64.8%) patients had grade 1-2 skin toxicities and only 2 patients had grade 3 skin toxicity (3.7%). One patient developed pneumonitis and none had cardiac or esophageal toxicity (**Table 3**). Regarding late toxicities, we did not observe non-cutaneous toxicities. Grade 0 and grade 1 cutaneous toxicities were observed in 28 and 16 patients (56% and 32%), respectively. Grade ≥2 toxicities concerned only skin (n=6 patients for any late skin toxicity; 12%) (**Table 3**). The main grade 3 toxicities were fibrosis and shrinking.

**Table 3:** Acute and late radiation-related toxicities.

CTCAE grade		Per patient (n=54), number (%)				Per breast (n=108), number (%)			
		0	1	2	3	0	1	2	3
**Acute** **toxicities**	Skin	17 (31.5%)	18 (33.3%)	17 (31.5%)	2 (3.7%)	34 (31.5%)	36 (33.3%)	34 (31.5%)	4 (3.7%)
	Lung	53 (98.1%)	1 (1.9%)	–	–	na	na	na	na
	Esophagus	45 (83.3%)	6 (11.1%)	3 (5.6%)	–	na	na	na	na
	Heart	54 (100%)	–	–	–	na	na	na	na
**Late** Toxicities*	Skin	28 (56%)	16 (32%)	5 (10%)	1 (2%)	59 (59%)	31 (31%)	9 (9%)	1 (1%)
	*Fibrosis*	32 (64%)	2 (4%)	4 (8%)	1 (2%)	66 (66%)	26 (26%)	7 (7%)	1 (1%)
	*Hyperpigmentation*	48 (96%)	2 (4%)	–	–	94 (94%)	6 (6%)	–	–
	*Shrinking*	44 (88%)	2 (4%)	4 (8%)	–	91 (91%)	3 (3%)	6 (6%)	–
	*Telangiectasia*	49 (98%)	1 (2%)	–	–	99 (99%)	1 (1%)	–	–
	*Breast edema*	46 (92%)	4 (8%)	–	–	92 (92%)	8 (8%)	–	–
	Lung	50 (100%)	–	–	–	na	na	na	na
	Esophagus	50 (100%)	–	–	–	na	na	na	na
	Heart	50 (100%)	–	–	–	na	na	na	na

na, not applicable. *Late toxicities were performed on 50 patients and 100 breasts, as 4 patients died or were lost to follow-up within 6 months after radiation therapy protocol.

Both neoadjuvant and adjuvant chemotherapy protocols were not associated with higher risk of acute and late toxicities (p=NS). Univariate analyses showed that PTV volume was associated with grade ≥2 acute skin toxicities (OR 1.0015 [95% CI 1.001-1.002]; p-value <0.05). Multivariate analysis performed with surgery type, HER2 amplification, SBR grade, histology and PTV as variables confirmed the association between grade ≥2 acute skin toxicities and PTV (OR 1.01 [95% CI 1.001-1.02]; p-value <0.05) and HER2 amplification (OR 4.22 [95% CI 1.012-17.65]; p-value <0.05).

Similarly, univariate analysis showed a significate association between PTV and grade ≥2 late skin toxicities (OR 1.001 [95% CI 1.0002-1.003]; p-value <0.05). Multivariate analysis could not be performed because of the small number of patients with grade ≥2 late skin toxicity. By matching follow-up data, no significant association was found between acute and late skin toxicities (p-value = 0.12).

### Outcomes and survival

**Figure 1:**
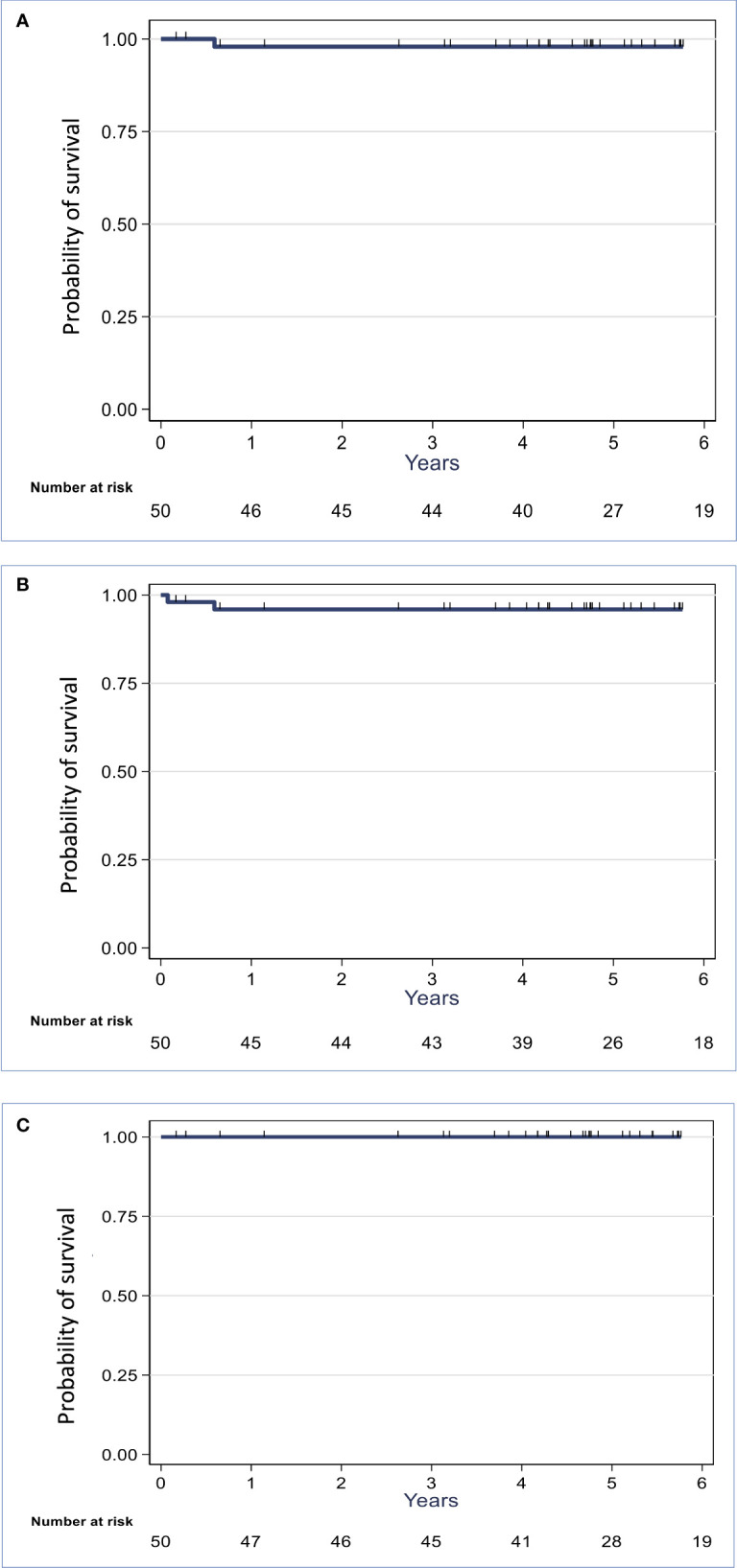
Kaplan-Meier locoregional-free **(A)**, recurrence-free **(B)** and overall **(C)** survival curves for the patients with initially non-metastatic synchronous bilateral breast cancer (n=50).

When considering the cohort of patients in the curative setting (n=50), the median LRFS, RFS and OS were not reached at the endpoint date. The 5-year LRFS, RFS, and OS rates were 98% [95% CI 86.12-99.70%], 96% [95% CI 84.63-98.96%], and 100%, respectively (**Figure 1**). Two patients had disease recurrence: one developed pleural metastases and one nodal disease (one positive axillary node at 1 year after the initial treatment). The patient with nodal recurrence had multifocal right breast cancer (pT2N2M0) and underwent lumpectomy - axillary node dissection followed by adjuvant chemotherapy, VMAT (breast/SIB + regional lymph nodes), and hormone therapy. The nodal recurrence was managed by lymph node excision. These two local recurrences occurred outside the VMAT irradiation field, showing a good local control.

In the whole cohort (n=54), the median RFS and OS were not reached at the endpoint date. The 5-year RFS and OS rates were 88.5% [95% CI 76.12-94.66%] and 93.54% [95% CI 81.16%-97.89%], respectively (**Supplementary Data, Figure 3**). Noteworthy, metastatic progression occurred in four patients, of whom three already had metastases at diagnosis. Among these four metastatic patients, three died because of BC progression.

## Discussion

To the best of our knowledge, this is the largest (n=54 patients) and longest study (median follow-up = 63 months) to evaluate VMAT efficiency and tolerance in patients with SBBC. This cohort included all consecutive patients, regardless of the presence of metastases, to reach a higher number of patients.

Firstly, our dosimetric data -performed on the 20 first patients included- confirmed that VMAT for SBBC is technically feasible, with at least 98% of PTV encompassed by more than 95% of the prescribed dose for breast, supraclavicular lymph nodes and boost PTV coverage, in agreement with the study by Nicolini et al. (7). Target volume coverage, planning objectives, and constraints differ among centers. In our study, target volumes were outlined according to the RTOG definition to use breast PTV-eval for the dosimetry study. This definition allows an inter-patient comparison of breast dose distribution regardless of the patients’ anatomy. When locoregional lymph nodes were not irradiated, the lung volume encompassed by the isodoses 5Gy and 20Gy was lower in the study by Nicolini et al. than in our study (SBBC with locoregional lymph node irradiation): 58% versus 77-79% (V5Gy) and 9.7–10.3% versus 17-18% (V20Gy) and the mean heart dose was slightly higher in our study (8.8Gy *versus* 6.0Gy in the study by Nicolini et al.) (7). While mean heart dose is reported to be around 8 Gy with IMC irradiation for unilateral BC (*versus* 12.5Gy in our study), we obtained lower values than what described in the literature for similar patients with SBBC (16). This indicates that adequate target volume coverage is possible with reduced heart (10.7Gy in our study *versus* 16.5Gy in the study by Lee et al.) and lung exposure (V20Gy and V30Gy of 17% and 8-9% in our study *versus* 23% and 12% in the study by Lee et al.) (6).

Secondly, regarding safety, VMAT exhibits an interesting safety profile. Indeed, only two (3.7%) patients had acute grade 3 skin toxicity and only one patient (2%) developed late grade 3 skin toxicity (breast fibrosis). Although heart and lungs are exposed to non-negligible dose of radiation, we did not observe all along the follow-up (with a median of 63 months) any clinical cardiac, esophageal or respiratory consequences in our cohort, even in the context of IMC irradiation. Interestingly, grade ≥2 acute skin toxicity occurrence was associated with PTV (volume), in accordance with previous VMAT data in patients with unilateral BC (17, 18). However, compared with the study by Fiorentino et al. (n=16 patients and 24 months of follow-up) (13), we observed fewer acute grade 1 and 2 skin toxicities (33.3% and 31.5% in our study *versus* 72% and 24% in the study by Fiorentino et al.). Furthermore, Fiorentino *el at.* determined late lung toxicity (*i.e.* lung fibrosis) by CT imaging (allowing the detection of subclinical toxicity), but did not record any late cardiac toxicity event, at least clinically (14). We found an association between PTV and acute and late grade ≥2 skin toxicities, but the odds ratio values were close to 1, suggesting a low clinical significance. Regarding the higher risk of skin toxicity associated with HER-2 amplification, one of the hypotheses is the confounding effect of trastuzumab, which has been proposed to promote radiosensitization (19).

Thirdly, VMAT for SBBC exhibits relevant treatment efficiency. In patients with local/locoregional SBBC at diagnosis (n=50), the 5-year OS rate was 100%. Furthermore, although two recurrences were recorded, none was in the irradiation field, showing that multimodal treatment with VMAT allows complete local disease control, as reported by Fiorentino et al. (100% survival and 100% local disease control at 24 months of follow-up) (14). Interestingly, although IMC PTV coverage could appear as suboptimal, we did not observe any difference regarding PFS and OS in patients with and without IMC node irradiation. This is probably due to the CBCT-based repositioning of patients at each session. Furthermore, although the dose volume distribution profiles were different between patients with and without IMC node irradiation, PFS and OS were not different between these patients. While consistent with the literature, it is important to point out that the present study included 50 patients (*versus* 9 to 25 in previous studies) and had a longer follow-up (63 months *versus* 10 to 36 months in previous studies) (13–15).

The main limitation of our study is its monocentric design. However, as SBBC is a rare event and as dosimetric parameters, target volume coverage, planning objectives and constraints differ according to the center, this monocentric study allowed collecting homogeneous data and obtaining a more reliable analysis. Another limitation is its retrospective design; nevertheless, data were prospectively collected, thus reducing the putative bias. On the other hand, SBBC rarity and the relatively large cohort of patients allow considering the findings of some value.

In conclusion, this study confirmed that VMAT for SBBC is technically feasible and exhibits an interesting efficiency/tolerance profile. Furthermore, the large size of our cohort and the longer follow-up compared to previous studies allowed showing that VMAT has favorable safety and efficiency profiles, and thus is suitable for SBBC management.

## Data availability statement

The raw data supporting the conclusions of this article will be made available by the authors, without undue reservation.

## Ethics statement

The studies involving human participants were reviewed and approved by Institut régional du Cancer de Montpellier local Ethics Committee. Written informed consent for participation was not required for this study in accordance with the national legislation and the institutional requirements.

## Author contributions

All the authors were involved in the conception/design of the work and provide approval for publication of the content more specifically. SQ, PF and CB contributed to drafting the work. All authors contributed to the article and approved the submitted version.

## Funding

Institut régional du Cancer de Montpellier, France.

## Acknowledgments

The authors thank Elisabetta Andermarcher for English editing and Océane Massot for assistance in statistical programming.

## Conflict of interest

The authors declare that the research was conducted in the absence of any commercial or financial relationships that could be construed as a potential conflict of interest.

## Publisher’s note

All claims expressed in this article are solely those of the authors and do not necessarily represent those of their affiliated organizations, or those of the publisher, the editors and the reviewers. Any product that may be evaluated in this article, or claim that may be made by its manufacturer, is not guaranteed or endorsed by the publisher.
