# Supplementary material for: Efficacy, safety, and feasibility of volumetric modulated arc therapy for synchronous bilateral breast cancer management
Source: Front Oncol. 2022 Aug 18;12:967479. doi: 10.3389/fonc.2022.967479 (PMC9436014; doi:10.3389/fonc.2022.967479)
Supplement: Supplementary file 1 [file DataSheet_1.pdf]

Supplementary data

Patient positioning/immobilization for VMAT and anatomy data acquisition

All patients underwent 3D-computed tomography (RT 16, GE, Milwaukee) without contrast enhancement in free breathing conditions (maximum slice thickness: 2.5 mm every 2.5 mm). Patients were in supine position, both arms up and behind the head. Anatomy image acquisition was from 2 cm above the clavicle to the infra-mammary fold, including the entire lung volume. All patients used a custom positioning cushion (Moldcare) and an upper extremity positioning system (Posirest, CIVCO, The Neverland) for positioning accuracy. Before this study, thoracic motion in breath-free conditions was assessed in 50 patients by 4D-CT image acquisition, leading to the decision of not using phase gating during irradiation (unpublished data: mammary gland moved ~1 mm in the craniocaudal direction during normal breathing). Before each VMAT session, cone-beam CT (CBCT) with a reduced dose, following the ALARA methodology, was performed to ensure good patient positioning.

Target volume definition and organs at risk

The gross tumor volume was considered equal to the Clinical Target Volume (CTV). Mammary gland and boost CTVs were delineated as previously described [2]. The CTVs of chest wall, supra- and infra-clavicular lymph nodes, and internal mammary chain (IMC) lymph nodes were defined according to the Radiation Therapy Oncology Group (RTOG) definition [11]. For optimization and dose reporting, the CTV was cropped 5mm under the skin and a new structure was defined: "CTV_opt". As no breast motion was observed in a previous study, no internal target volume was required. The Planning Target Volume (PTV) was defined as the CTV with an additional margin of 7mm with cropping at 5mm under the skin. The PTV-eval was defined as the breast volume the posterior limit of which was located to the rib anterior surface (boney thorax and lung excluded). According to the RTOG 10-05 guidelines, the breast PTV-eval was not used for beam aperture generation. To analyze the quality of dose distribution (breast volume coverage), the mammary gland PTV - boost PTV was calculated. Lungs, heart, esophagus, trachea, liver, thyroid and spinal cord were considered as the OAR. No bolus was allowed to prevent skin toxicity.

Dose prescription and treatment planning

In case of axillary lymph node involvement, supra- and infra-clavicular and IMC lymph nodes were treated. In patients with pN0 SBBC, only breast was considered for irradiation; boost indication was left to the radiation oncologist's discretion and was simultaneously integrated to the breast field. A total dose (TD) of 52.2Gy in 29 fractions was delivered to the breast and IMC PTVs [i.e., biological effective dose at 2 Gy (BED2) = 50Gy /25 fractions]; a TD of 49.3Gy in 29 fractions to the supra- and infra-clavicular lymph node PTV (BED2=45Gy/25 fractions); and a TD of 63.22Gy in 29 fractions to the boost PTV (BED2= 66Gy/33 fractions). BED2 was calculated as previously described (15). If possible, 95% of the prescribed dose had to encompass at least 95% of the breast PTV-eval, at least 99% of the CTV_opt, and 90% of each PTV. Dose exposure to OAR should respect the following constraints (volume encompassed by the isodose xGy, VxGy; dose received by not more than x% of the volume, Dx%; mean dose, Dmean): i) for each lung: V20Gy <22%, V30Gy <10%; Dmean <13Gy; V10Gy and V5Gy as low as possible; ii) for heart: Dmean as low as possible (<10Gy); iii) for thyroid, esophagus, trachea and liver: minimization of radiation exposure; and iv) for spinal cord: D1% <40 Gy. VMAT plans were optimized using 2 to 4 partial arcs (range, ± 270° to 180°; clockwise and counter clockwise) that shared the same isocenter with a collimator rotation of ± 10°. Treatment plan optimization was performed using the Varian Eclipse treatment planning system (version 10.0.28) with 6 MV photon beams from a Varian Truebeam Accelerator equipped with a 120-leaf Millenium Multileaf Collimator. The anisotropic Analytical Algorithm (AAA) photon algorithm was used for dose calculation with a grid of 2.5 mm. The maximum dose rate was fixed to 400 MU/min, and field opening varied from 15 to 17 cm in the X jaw direction with complete asymmetry.

REFERENCES

1. Fowler JF. 21 years of Biologically Effective Dose. *BJR* (2010) 83:554–568. doi: 10.1259/bjr/31372149
2. Charaghvandi RK, den Hartogh MD, van Ommen A-MLN, de Vries WJH, Scholten V, Moerland MA, Philippens MEP, Schokker RI, van Vulpen M, van Asselen B, et al. MRI-guided single fraction ablative radiotherapy for early-stage breast cancer: a brachytherapy versus volumetric modulated arc therapy dosimetry study. *Radiother Oncol* (2015) 117:477–482. doi: 10.1016/j.radonc.2015.09.023

Dosimetric analysis of the first 20 treatment plans.

Supplementary Table 1 summarizes the analysis of the treatment plans based on the dose volume histograms. **Supplementary Figure 1** shows the mean distribution volumes for the various target volumes and **Supplementary Figure 2** shows the mean distribution volumes for the organs at risk.

From a quantitative point of view, the breast PTV-eval coverage was adequate because at least 95% of the volume was encompassed by 98.9% of the prescribed dose (Supplementary Fig. 1). At least 95% of the breast PTV was encompassed by 91% of the prescribed dose (Supplementary Fig. 1). The coverage of the supraclavicular lymph node PTV and boost PTV (Supplementary Fig. 1) also was adequate (95% of the prescribed dose encompassed at least 90% of each PTV). Conversely, the IMC lymph node PTV coverage was not enough because less than 90% of the PTV was encompassed by 95% of the prescribed dose. However, the IMC lymph node CTV coverage was correct (>95% of the volume was encompassed by 95% of the prescribed dose) (Supplementary Fig. 1). The exposure of organs at risk (OAR) fitted with the dosimetric constraints in patients with and without lymph node irradiation. The mean lung dose was 12.3Gy (range: 7.7 – 18.7), and the V30Gy and V20Gy were <10% and <20%, respectively. Lung exposure was comparable on both sides (red curves in Supplementary Fig. 2a and 2d). The mean heart dose was 10.7Gy (range: 6.2 – 22.3) and the V30Gy was 4.3 ± 1.7 Gy (red curve in Supplementary Fig. 2b). To analyze the effect of lymph node area irradiation on OAR exposure, a subgroup analysis was performed in patients with (green) and without node irradiation (blue). No difference was observed for lung exposure (Supplementary Fig. 2a and 2d). Conversely, heart, esophagus, spinal cord, and trachea exposure was lower in the group without node irradiation. For instance, the D1cm^3 for spinal cord was 12.5Gy and 34.3Gy with and without node irradiation, respectively (Supplementary Fig. 2c). Similarly, spinal cord exposure was lower in the group without node irradiation (V10Gy: 17.7±11.4 cm^3 and 2.7±3.7 cm^3 in the group with and without node irradiation, respectively). The trachea V40Gy and V20Gy were 0 and 0.7±1.2 cm^3 in the group without node irradiation and 5.0±3.9 and 12.9±6.1 cm^3 in the group with node irradiation (Supplementary Fig. 2f). The heart V30Gy also was lower in the group without than with node irradiation (1.3±1.3% versus 7.4±9.1%) (Supplementary Fig. 2b). The mean heart dose was 8.8±1.8 Gy in patients who underwent breast irradiation alone (regardless of the breast side). This value increased by 4 Gy in the group with node irradiation (12.5±5.8 Gy). In esophagus, the D1 cm^3 was strongly decreased in patients without node irradiation (15.8 Gy compared with 44.5 Gy in patients with node irradiation).

Patients follow-up:

Supplementary Figure 3 shows the progression free (3s-a) and overall (3s-b) survival curves for the whole cohort (n=54).

				CTV_opt		PTV		OAR	
				Dosimetric constraints	Dose Volume Histogram Results	Dosimetric constraints	Dose Volume Histogram Results	Dosimetric constraints	Dose Volume Histogram Results
Target volumes	Breast	V95 % (%, SD)	Right	99%	98.9 (0.9)	90%	92.0 (6.3)	-	-
		V95 % (%, SD)	Left		98.9 (1.1)		91.5 (6.2)	-	-
	Internal mammary chain	V95 % (%, SD)	Right	95%	98.0 (5.2)	95%	80.5 (15.8)	-	-
		V95 % (%, SD)	Left		98.8 (2.4)		84.7 (17.0)	-	-
	Supraclavicular nodes	V95 % (%, SD)	Right	95%	99.7 (0.6)	95%	94.3 (5.0)	-	-
		V95 % (%, SD)	Left		99.9 (0.2)		96.4 (2.7)	-	-
	Boost	V95 % (%, SD)	Right	95%	99.4 (1.6)	95%	94.0 (7.8)	-	-
		V95 % (%, SD)	Left		98.9 (2.3)		92.3 (12.8)	-	-
Organs at Risk	Lung	Mean (Gy, SD)	Right	-	-	-	-	< 13Gy	12.7 (2.2)
			Left	-	-	-	-		12.0 (2.5)
		V30Gy (%, SD)	Right	-	-	-	-	< 10%	8.9 (2.9)
			Left	-	-	-	-		8.3 (3.9)
		V20Gy (%, SD)	Right	-	-	-	-	< 20%	18.6 (4.9)
			Left	-	-	-	-		17.1 (6.1)
		V10Gy (%, SD)	Right	-	-	-	-	< 50%	43.6 (12.5)
			Left	-	-	-	-		38.9 (11.4)
		V5Gy (%, SD)	Right	-	-	-	-	< 80%	79.6 (14)
			Left	-	-	-	-		77.7 (16.1)
	Heart	Mean dose (Gy, SD)	-	-	-	-	-	<10Gy	10.7 (4.6)
		V5Gy (%, SD)	-	-	-	-	-	-	79.9 (18.8)
		V30Gy (%, SD)	-	-	-	-	-	<5%	4.3 (1.7)
		V40Gy (%, SD)	-	-	-	-	-	0%	0.5 (1.1)

Supplementary table 1: Clinical Target Volume (CTV) and Planning Target Volume (PTV) coverage, and Organ At Risk (OAR) sparing.

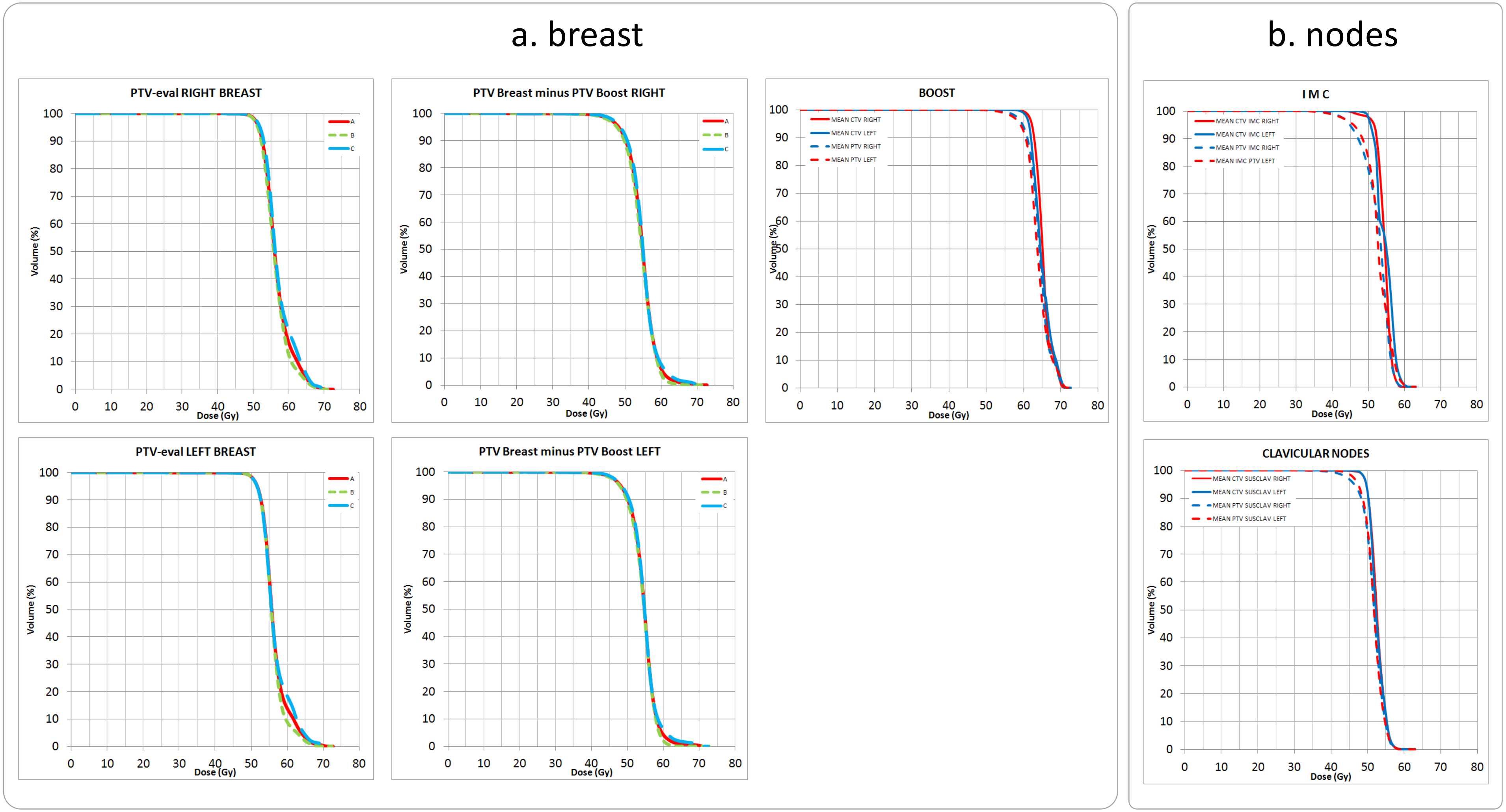

Supplementary figure 1: dose volume distribution for breast (a) and nodes(b) from the 20 first patients from the cohort

Color legends: in red (A), mean dose of target volume for the entire cohort of patients; in green (B), mean dose of target volume for patients who underwent nodes area irradiation; in blue (C), mean dose of target volume for patients who only underwent breast and boost irradiation without nodes area irradiation. Abbreviations: IMC = internal mammary chain; PTV = planning target volume.

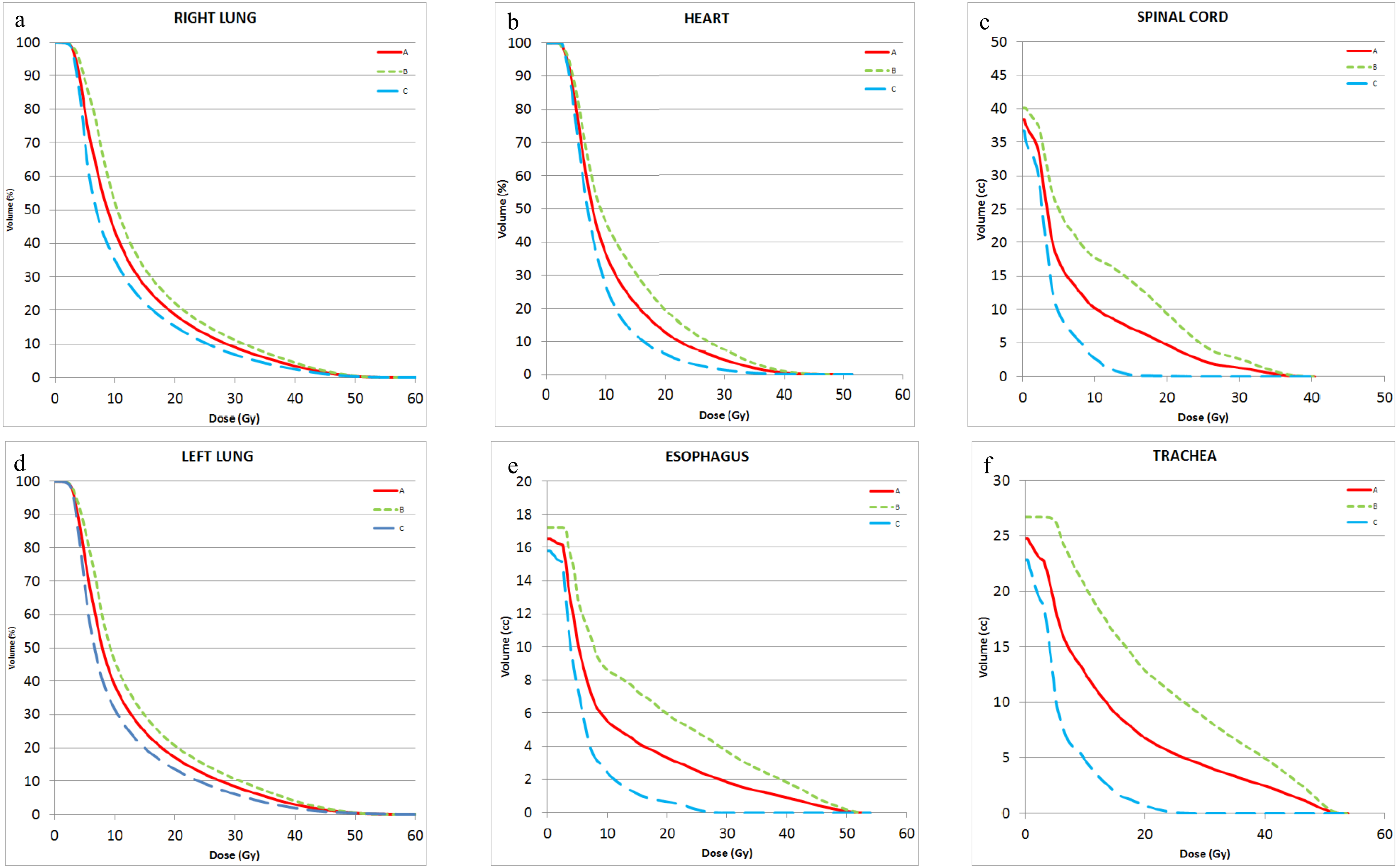

Supplementary figure 2: dose volume distribution of organs at risk = (a) and (b) lungs ; (c) heart; (d) esophagus ; (e) spinal cord; and (f) trachea.

Color legends: in red (A), mean dose of target volume for the entire cohort of patients; in green (B), mean dose of target volume for patients who underwent nodes area irradiation; in blue (C), mean dose of target volume for patients who only underwent breast and boost irradiation without nodes area irradiation.

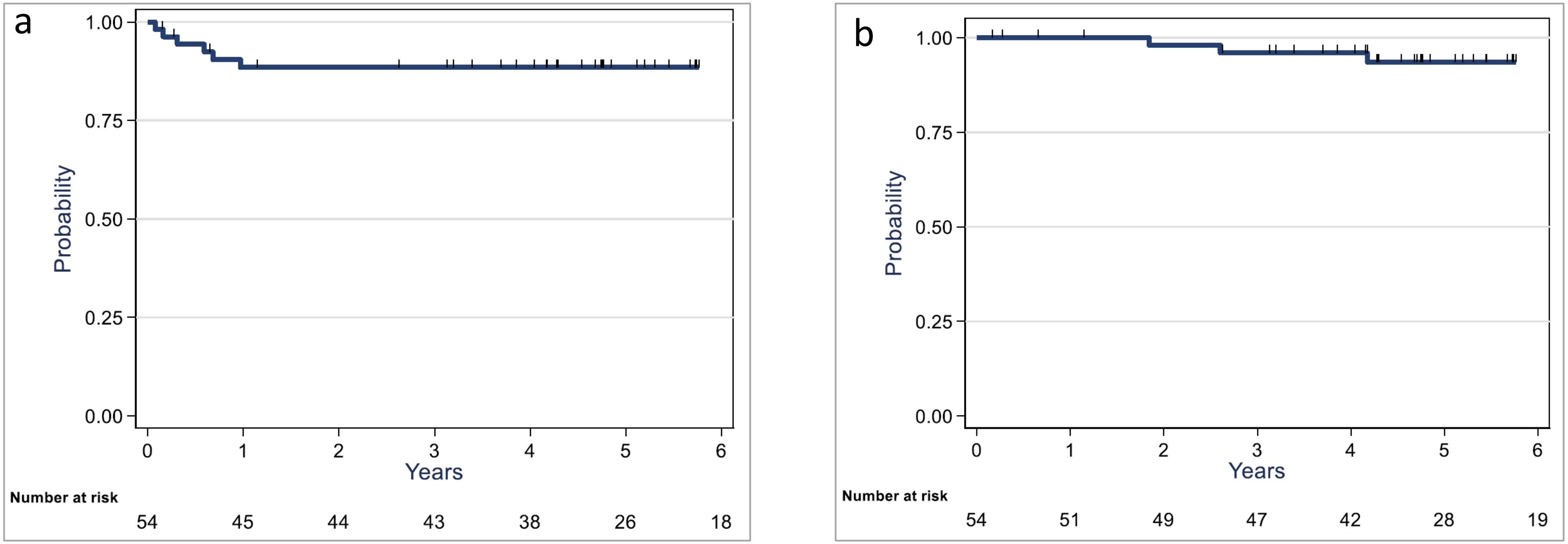

Supplementary figure 3: Kaplan-Meier progression-free (a) and overall (b) survival curves for the whole cohort (n=54).
